# SOX2 promotes vasculogenic mimicry by accelerating glycolysis via the lncRNA AC005392.2-GLUT1 axis in colorectal cancer

**DOI:** 10.1038/s41419-023-06274-1

**Published:** 2023-12-04

**Authors:** Shimiao Huang, Xuan Wang, Yin Zhu, Yadong Wang, Jiaxuan Chen, Haoxuan Zheng

**Affiliations:** 1 Guangdong Provincial Key Laboratory of Gastroenterology, Department of Gastroenterology, Nanfang Hospital, Southern Medical University, 510515 Guangzhou, China; 2 School of Laboratory Medicine and Biotechnology, Southern Medical University, 510515 Guangzhou, China; 3 State Key Laboratory of Organ Failure Research, Guangdong Key Laboratory of Viral Hepatitis Research, Guangdong Institute of Liver Diseases, Department of Infectious Diseases and Hepatology Unit, Nanfang Hospital, Southern Medical University, 510515 Guangzhou, China

**Keywords:** Metastasis, Cell migration, Prognostic markers, Diagnostic markers

## Abstract

Vasculogenic mimicry (VM), a new model of angiogenesis, fulfills the metabolic demands of solid tumors and contributes to tumor aggressiveness. Our previous study demonstrated the effect of SOX2 in promoting VM in colorectal cancer (CRC). However, the underlying mechanisms behind this effect remain elusive. Here, we show that SOX2 overexpression enhanced glycolysis and sustained VM formation via the transcriptional activation of lncRNA AC005392.2. Suppression of either glycolysis or AC005392.2 expression curbed SOX2-driven VM formation in vivo and in vitro. Mechanistically, SOX2 combined with the promoter of AC005392.2, which decreased H3K27me3 enrichment and thus increased its transcriptional activity. Overexpression of AC005392.2 increased the stability of GLUT1 protein by enhancing its SUMOylation, leading to a decrease in the ubiquitination and degradation of GLUT1. Accumulation of GLUT1 contributed to SOX2-mediated glycolysis and VM. Additionally, clinical analyses showed that increased levels of AC005392.2, GLUT1, and EPHA2 expression were positively correlated with SOX2 and were also associated with poor prognoses in patients with CRC. Our study conclusively demonstrates that the SOX2-lncRNA AC005392.2-GLUT1 signaling axis regulates VM formation in CRC, offering a foundation for the development of new antiangiogenic drugs or new drug combination regimens.

## Introduction

Colorectal cancer (CRC) is the third most common malignancy worldwide, with both high morbidity and mortality rates [1]. Metastasis is reported as a primary driver of CRC-related mortality [2, 3]. Activation of angiogenesis is an essential hallmark of cancer, and is required for invasive tumor growth and metastasis [4]. As diagnostic and therapeutic strategies progress rapidly, especially in the application of immunotherapy and anti-angiogenesis therapy [5], the overall survival rate of CRC has improved. VEGF/VEGFR is the primary drug target for anti-angiogenic therapeutic applications. Unfortunately, the therapeutic benefits of anti-VEGF/VEGFR therapy did not reach the high expectations which were anticipated based on preclinic studies [6, 7]. In addition to the unsatisfactory clinical efficacy, it is also associated with significant side effects and drug resistance. Thus there is an important need to identify novel drug targets based on undiscovered mechanisms to try and combat tumor angiogenesis.

Vasculogenic mimicry (VM) is a new model of tumor microcirculation that is distinct from classical tumor angiogenesis. In VM, tumor cells form blood vessels and provide sufficient blood supply independent of endothelial cells [8]. VM occurs in certain highly aggressive malignancies, and is closely associated with poor clinical prognoses. Of note, VM is VEGF-independent and therefore capable of mediating tumor vascularization despite VEGF-inhibition, which is likely the cause for failure in some antiangiogenic therapy. Thus VM has become one of the more promising potential targets for anticancer therapy. Although anti-VM strategies are under clinical development, the fundamental mechanisms underlying the action of VM are largely unknown. Our previous study showed that Sex-determining region Y-box2 (SOX2), a master regulator of embryonic development, drives tumor growth and invasion [9], promotes VM formation in CRC [10], however, the underlying mechanism of action remains unclear.

Glycolysis has been shown to promote angiogenesis by satisfying the glycolytic requirements of vascular endothelial cells and generating an acidic environment [11]. However, the involvement of glycolysis in VM formation of CRC cells is not well-characterized. Moreover, SOX2 is known to increase glycolysis in prostate cancer cells [12], although the association between glycolysis and SOX2-mediated VM formation remains unclear. Clarification of the underlying molecular mechanism may help identify potential diagnostic biomarkers and potential therapeutic targets.

Long non-coding RNAs (lncRNAs) represent a heterogeneous class of transcripts of greater than 200 nucleotides (nt), with little or no protein-coding potential [13]. Mounting evidence suggests that lncRNAs play a significant role in the carcinogenesis and progression of a wide variety of human malignancies via regulating multiple biological processes, including metabolism, invasion, and metastasis [14]. Moreover, lncRNAs have been shown to participate in SOX2-induced proliferation and metastasis in non-small-cell lung carcinoma [15]. However, the correlation between lncRNAs and SOX2 in CRC is rarely reported and the effect of lncRNAs on SOX2-induced VM formation remains unclear.

In this study, we unravel a molecular pathway responsible for the SOX2-mediated promotion of VM formation in CRC cells via the SOX2-lncRNA AC005392.2-GLUT1 signaling axis. Our findings may help identify novel diagnostic biomarkers and provide a promising strategy for treating VM formation through the inhibition of glycolysis in CRC and other types of VM-associated cancers.

## Materials and methods

The reagents of our research are listed in Table S1. A detailed method is available in the Supplemental Materials.

## Results

### SOX2 promotes VM by enhancing glycolysis in CRC cells

**Fig. 1:**
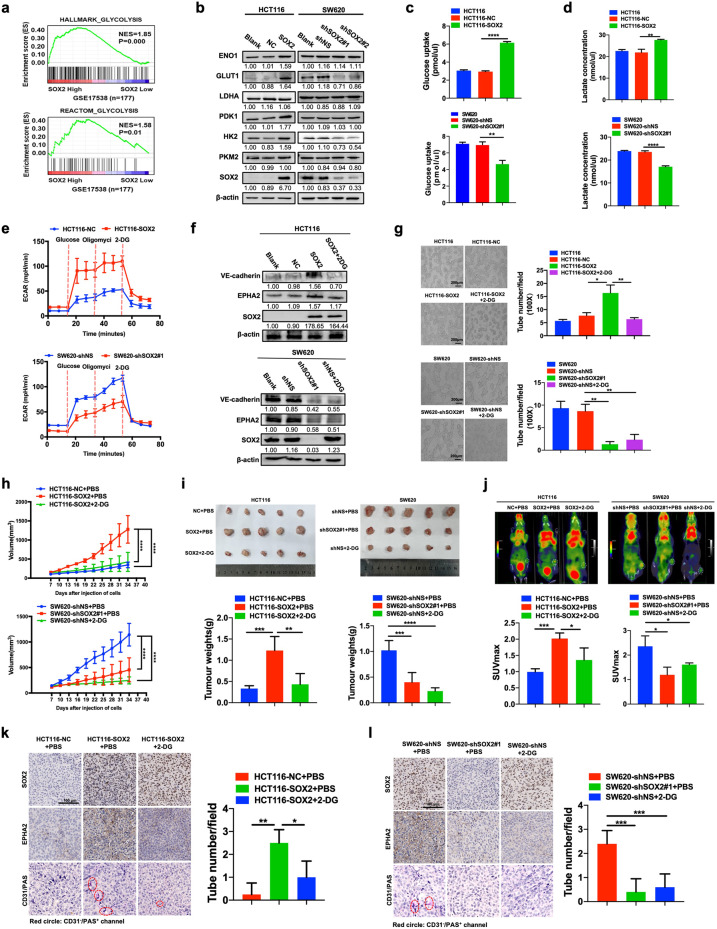
SOX2 promotes vasculogenic mimicry in CRC cells by stimulating glycolysis in vitro and in vivo. **a** GSEA showing that increased expression of SOX2 was positively correlated with glycolysis in gene expression profiles for patients with CRC (TCGA, *n* = 177). **b** Western blotting examining the expression of SOX2 and glycolytic molecules in HCT116 and SW620 cells after a 72 h transfection with a SOX2 clone or SOX2 shRNA. **c**, **d** The effect of SOX2 on glucose consumption (**c**) and lactate production (**d**) was assessed using fluorescence-based kits in HCT116 and SW620 cells (mean ± SD; *n* = 3, two-tailed Student’s *t* test). **e** The effect of SOX2 on ECAR was measured using a Seahorse XF assay in HCT116 and SW620 cells (mean ± SD; *n* = 3). **f**, **g** HCT116 and SW620 cells were transfected for 72 h using a SOX2 clone or SOX2 shRNA, then treated with 2-DG (3 mM) for 24 h. Western blotting was performed to analyze the indicated proteins (**f**). A tube formation assay was performed to assess VM formation (**g**, Scale, 200 μm; mean ± SD; *n* = 3, two-tailed Student’s *t* test). **h** Plots of tumor volumes, measured every three days (mean ± SD; *n* = 5, two-way ANOVA test). **i** Representative tumor images and a summary of tumor weight data. Tumors were harvested after mice were euthanized (mean ± SD; *n* = 5, two-tailed Student’s *t* test). **j** ^18^F-FDG uptake and SUVmax of xenografts were examined using PET/CT (mean ± SD; *n* = 3, two-tailed Student’s *t* test). **k**, **l** IHC staining of SOX2, EPHA2, and CD31/PAS in SOX2-overexpression HCT116 (**k**) and SOX2-knockdown SW620 xenografts (**l**, Scale, 100 μm). The number of VM structures (CD31−/PAS+) was calculated (mean ± SD; *n* = 4, two-tailed Student’s *t* test). *p < 0.05, **p < 0.01, ***p < 0.001, and ****p < 0.0001.

Our previous study revealed that SOX2 promotes VM formation in CRC cells [10], while more recent studies have reported that inhibition of glycolysis suppressed VM formation in nasopharyngeal carcinoma [16]. This prompted us to explore whether glycolysis was involved in SOX2-induced VM in CRC cells. We first investigated the regulation of SOX2 on glycolysis in CRC cells. Gene set enrichment analysis (GSEA) indicated that high expression of SOX2 was positively correlated with “HALLMARK_GLYCOLYSIS” and “REACTOM_ GLYCOLYSIS” gene signatures in CRC (GSE17538, *n* = 177) (Fig. 1a). Gene Ontology (GO) analysis of differential expression mRNA impaired by SOX2 illustrated that genes regulated by SOX2 overexpression were enriched in the glycolytic process, indicating there is an interaction between SOX2 expression and glycolysis (Fig. S1a). Then, qRT-PCR and western blotting were performed to evaluate the expressions of SOX2 in four human CRC cell lines, namely, HCT116, RKO, SW620 and LOVO. The results revealed that SOX2 was markedly elevated in SW620 cells and depressed in HCT116 cells at both the mRNA and protein levels (Fig. S1b). Of note, overexpression of SOX2 increased the expression of glycolytic molecules (Figs. 1b and S1c), glucose consumption (Fig. 1c), and lactate production (Fig. 1d) in HCT116 cells, while knockdown of SOX2 led to the opposite results in SW620 cells. Importantly, the results of the extracellular acidification rate (ECAR) assay further validated that SOX2 overexpression effectively enhanced glycolysis, whereas SOX2 depletion did the opposite (Fig. 1e). Taken together, these data demonstrate that SOX2 enhances glycolysis in CRC cells.

We then sought to investigate whether glycolysis functioned as a downstream component of SOX2 to promote VM in CRC cells. SOX2-overexpressing HCT116 cells were treated with 2-DG, an inhibitor of glycolysis. The results showed that inhibition of glycolysis partially abrogated SOX2-induced upregulation of VM-related molecules, including VE-cadherin and EPHA2 (Fig. 1f). Blockage of glycolysis impaired SOX2-mediated migration and VM formation in CRC cells (Figs. 1g and S1d). Overall, these findings demonstrate that SOX2 promotes VM formation in a glycolysis-dependent manner in CRC cells.

### SOX2-induced glycolysis facilitates VM formation in vivo

To further validate the involvement of glycolysis in SOX2-induced VM in vivo, xenograft models were established by subcutaneous inoculation with three types of stably transfected HCT116 cells or SW620 cells into the groin of 5-week-old nude mice. We consistently found that overexpression of SOX2 resulted in significant increases in tumor volume and weight, which was effectively abrogated by treatment with the glycolysis inhibitor, 2-DG (Fig. 1h, i). Moreover, Micro-PET imaging showed that overexpression of SOX2 led to increased uptake of ^18^F-Fluorodeoxyglucose (^18^F-FDG) and increased SUVmax, while a marked suppression was observed in mice treated with 2-DG (Fig. 1j). Xenograft tissues analyzed using immunohistochemistry (IHC) staining and western blotting revealed that SOX2 overexpression significantly increased the expression of VM-related molecules and VM formation in vivo, which was also effectively impaired by 2-DG treatment (Figs. 1k, l and S1e). Accordingly, either SOX2 depletion or inhibition of glycolysis by 2-DG led to a significant decrease in the tumor volume, tumor weight, uptake of ^18^F-FDG and SUVmax of xenografts, as well as VM formation in vivo (Figs. 1h–j, l and S1e). However, no significant difference was found between SOX2 depletion and inhibition of glycolysis. Collectively, these findings indicate that glycolysis is involved in SOX2-mediated VM formation in vivo.

### SOX2 enhances glycolysis by upregulating AC005392.2 expression in CRC cells

**Fig. 2:**
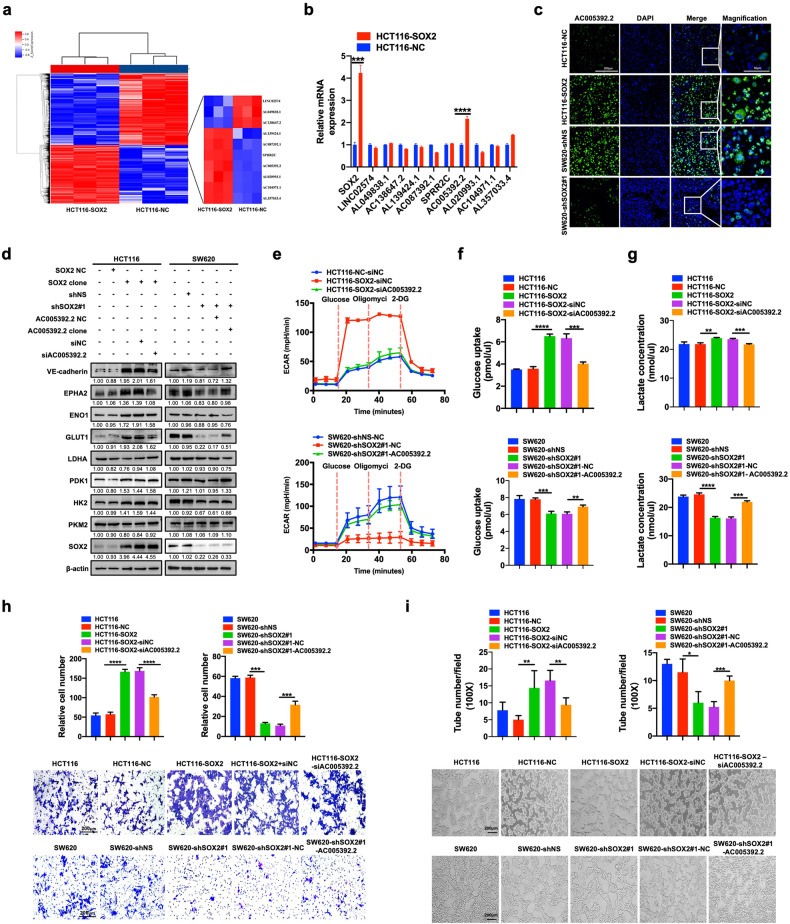
SOX2 enhances glycolysis by upregulating AC005392.2 expression in CRC cells. **a** Heatmap showing differentially expressed genes (DEGs, FDR < 0.05) in SOX2- overexpressing HCT116 cells based on lncRNA microarray analysis. **b** qRT-PCR examination of the top ten of most significant DEGs in panel A (mean ± SD; *n* = 3, two-tailed Student’s *t* test). **c** FISH assay detecting the expression and localization of AC005392.2 in tissues of HCT116 and SW620-based xenografts (Scale, 200 μm; inset: scale, 50 μm). **d** SOX2-overexpressing HCT116 cells or SOX2-deficient SW620 cells were transfected for 72 h with AC005392.2 siRNA or AC005392.2 clone plasmid. Western blotting was performed using the indicated antibodies. **e** The ECAR was examined in transfected HCT116 cells and SW620 cells using a Seahorse XF assay (mean ± SD; *n* = 3). **f**, **g** Glucose consumption (**f**) and lactate production (**g**) were assessed using fluorescence-based kits in transfected HCT116 cells and SW620 cells (mean ± SD; *n* = 3, two-tailed Student’s *t* test). **h**, **i** Transwell migration assay (**h**) and tube formation assay (**i**) were performed in transfected HCT116 cells and SW620 cells (Scale, 200 μm; mean ± SD; *n* = 3, two-tailed Student’s *t* test). *p < 0.05, **p < 0.01, ***p < 0.001, and ****p < 0.0001.

An increasing number of studies have demonstrated that lncRNAs play critical roles in tumor progression by regulating multiple biological processes, including glycolysis [17]. To investigate whether the potential lncRNAs contribute to SOX2-driven glycolysis and VM formation, lncRNA microarray analysis was performed in HCT116 cells before or after SOX2 overexpression. The results displayed significant differences in the gene expression profile in both cohorts (Fig. 2a). qRT-PCR was subsequently utilized to identify the top ten most significantly differential expression lncRNAs. Notably, lncRNA AC005392.2 was found to be markedly elevated in SOX2-overexpressing cells (Fig. 2b). Next, xenograft tissues were analyzed using fluorescence in situ hybridization (FISH), confirming the regulation of AC005392.2 by SOX2 in vivo (Fig. 2c). To further explore whether AC005392.2 potentiates SOX2-driven glycolysis and VM formation, we reversed AC005392.2 expression in HCT116 and SW620 cells stably transfected with SOX2 clone vector or SOX2 shRNA (Fig. S2a, b). The results revealed that reversing AC005392.2 expression partly blocked the effect of SOX2 on glycolytic molecules expression, glucose consumption, lactate production, and ECAR (Fig. 2d–g). Importantly, the expression of VM-related molecules and VM formation driven by SOX2 were also impaired by AC005392.2 dysregulation (Fig. 2d, h, i). Overall, these data prove that SOX2 promotes glycolysis and VM formation, at least partially relying on the upregulation of lncRNA AC005392.2 in CRC.

### SOX2 transcriptionally promotes lncRNA AC005392.2 expression by binding with and decreasing H3K27me3 enrichment on its promoter

**Fig. 3:**
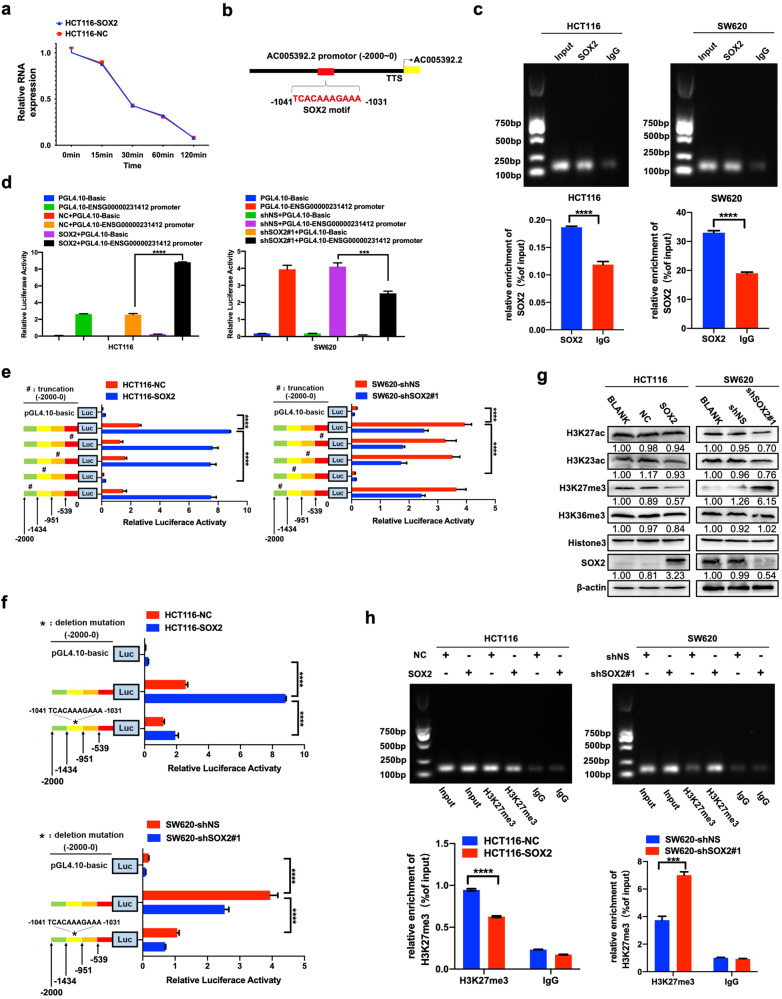
SOX2 decreases the enrichment of H3K27 methylation and transcriptionally activates AC005392.2 expression. **a** HCT116 cells were transfected for 72 h with a SOX2 clone and subjected to actinomycin D (2.5 μg/ml) treatment for the indicated times. qRT-PCR was performed to analyze the expression of AC005392.2 (mean ± SEM; *n* = 3). **b** A schematic map of potential SOX2-binding sites in the promoter of AC005392.2, shown according to the JASPAR database. **c** A ChIP assay was performed to confirm the interaction between the SOX2 and AC005392.2 promoter in HCT116 and SW620 cells after transfection for 72 h with SOX2 clones or SOX2 shRNA. An IgG antibody was used as a negative control (mean ± SD; *n* = 3, two-tailed Student’s *t* test). **d** AC005392.2 promoter-driven luciferase activity was assessed in HCT116 and SW620 cells transfected with a SOX2 clone or SOX2 shRNA using a luciferase reporter assay (mean ± SD; *n* = 3, two-tailed Student’s *t* test). **e**, **f** Luciferase activity of the AC005392.2 promoter was examined in HCT116 cells and SW620 cells transfected with a truncated AC005392.2 promoter (**e**) or deletion mutant of the AC005392.2 promoter (**f**, mean ± SD; *n* = 3, two-tailed Student’s *t* test). **g** Western blotting examining the expression of histone methylation and acetylation modification in SOX2-overexpressing HCT116 and SOX2-depleted SW620 cells. **h** A ChIP assay identifying the expression of H3K27me3 at the promoter of AC005392.2 in SOX2-overexpressing HCT116 and SOX2-depletion SW620 cells (mean ± SD; *n* = 3, two-tailed Student’s *t* test). ***p < 0.001, and ****p < 0.0001.

We then sought to determine whether the promotion of SOX2 on lncRNA AC005392.2 expression occurred during transcriptional activation or post-transcriptional regulation. First, we found that SOX2 overexpression had little effect on the half-life of AC005392.2 mRNA (Fig. 3a). Of note, a potential SOX2-binding site in the promoter of AC005392.2 (−1031 ~ −1041, upstream of the transcriptional starting site, TSS) was found using the JASPAR database (Fig. 3b). Our results further confirmed the binding of SOX2 on the AC005392.2 promoter by ChIP assay and luciferase reporter assays (Fig. 3c, d). We next constructed different truncations of the AC005392.2 promoter (0 ~ −539, −540 ~ −951, −952 ~ −1434, −1435 ~ −2000). As expected, we found transcriptional activity was significantly decreased in the third truncated region of the AC005392.2 promoter (−952 ~ −1434, Fig. 3e), which contained the predicted binding site (−1031 ~ −1041). Furthermore, deletion of this sequence (−1031 ~ −1041) markedly restrained SOX2-induced luciferase activity of the AC005392.2 promoter (Fig. 3f). Collectively, these data suggest that SOX2 upregulates AC005392.2 expression, partially through binding to its promoter.

Additionally, recent studies have shown that aberrant expression of lncRNA can be partially attributed to abnormal epigenetic regulation [18]. H3K27me3 methyltransferase is commonly believed to act by repressing gene expression [19]. We found that the expression of H3K27me3 was decreased by SOX2 overexpression, without any significant concurrent changes in the expression of H3K27ac, H3K23ac, or H3K36me3 (Fig. 3g). Furthermore, SOX2 overexpression reduced H3K27me3 enrichment in the promoter of AC005392.2 by ChIP-qPCR assay and agarose gel electrophoresis, while SOX2 depletion did the opposite (Fig. 3h). Overall, these results indicate that SOX2 can directly bind with and suppress H3K27me3 enrichment on the promoter of AC005392.2, helping to transcriptionally promote its expression in CRC cells.

### AC005392.2 activates glycolysis by binding with and increasing the stability of GLUT1 protein

**Fig. 4:**
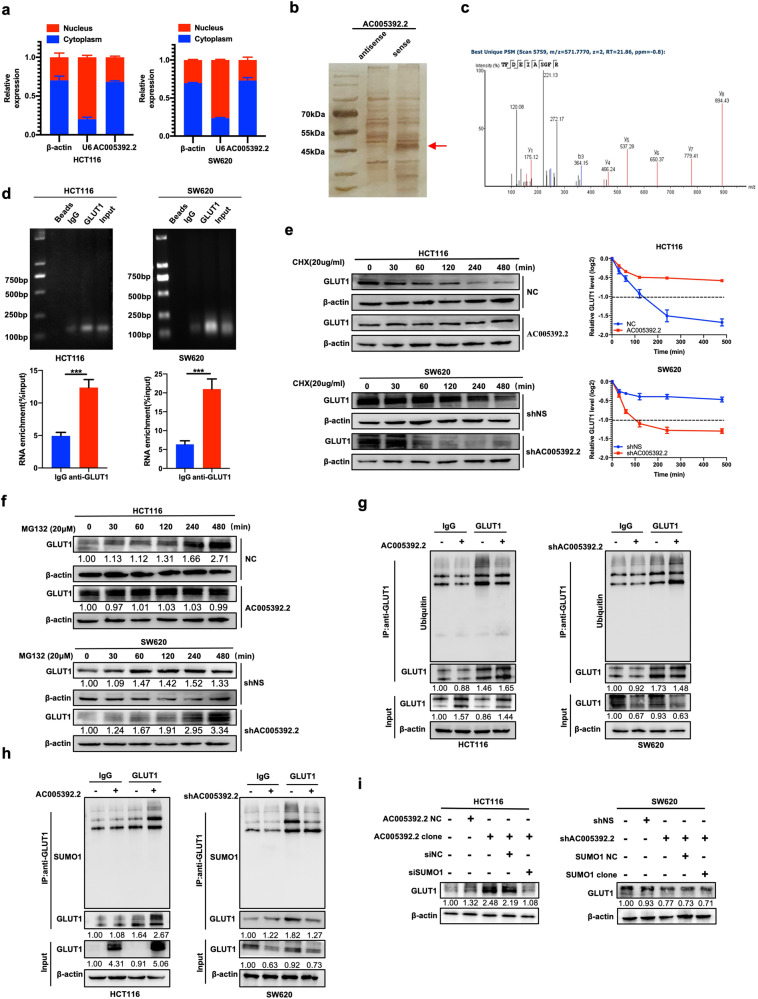
AC005392.2 activates glycolysis by binding and increasing the stability of GLUT1 protein. **a** qRT-PCR detection of AC005392.2 expression in the cytoplasmic and nuclear fractions of HCT116 and SW620 cells (mean ± SD; *n* = 3). **b** Silver staining showing the stripes pulled down by the sense and antisense AC005392.2 sequences. The arrow indicates the candidate stripes. **c** Unique peptides of GLUT1 were detected by mass spectrometry. **d** RIP assay showing the enrichment of AC005392.2 in the complex coprecipitated by anti-GLUT1 antibody in HCT116 and SW620 cells (mean ± SD; *n* = 3, two-tailed Student’s *t* test). **e**, **f** HCT116 and SW620 cells were transfected for 72 h with an AC005392.2 clone or AC005392.2 shRNA, then treated with 20 μg/ml cycloheximide (**e**) or 20 μM MG132 (**f**) for the indicated time. Western blotting was performed to analyze the expression of GLUT1. **g**, **h** Immunoprecipitation followed by western blotting was performed to analyze the ubiquitination (**g**) and SUMOylation (**h**) of GLUT1 in AC005392.2-overexpressing HCT116 and AC005392.2-knockdown SW620 cells. **i** Western blotting analysis of the protein levels of GLUT1 in HCT116 cells co-transfected with AC005392.2 clone and SUMO1 siRNA or in SW620 cells co-transfected with AC005392.2 shRNA and SUMO1 clone for 72 h. ***p < 0.001.

Given that the mechanisms of lncRNAs largely depend on their intracellular location [20], subcellular fractionation experiments were performed. We found that AC005392.2 was mainly present in the cytoplasm of CRC cells (Fig. 4a), indicating that AC005392.2 may exert its function at a post-transcriptional level. The results of silver staining revealed an increased level at the size of 45–55 kDa in the products pulled down by the full-length AC005392.2 sense sequence compared with the antisense sequence (Fig. 4b). Mass spectrometry analysis of these products showed that 206 proteins were pulled down by the AC005392.2 sense sequence and 283 proteins by the antisense sequence (Table S2). Glycolytic molecules, including alpha-enolase (ENO1), pyruvate kinase (PKM) fructose-bisphosphate aldolase A (ALDOA), and phosphoglycerate kinase 1 (PGK1), appeared in the products pulled down by both AC005392.2 sense and antisense sequence, while glucose transporter member 1 (GLUT1) was pulled down by only the AC005392.2 sense sequence (Fig. 4c and Table 1). Further results confirmed the interaction between GLUT1 and AC005392.2 by RIP assay and agarose gel electrophoresis (Fig. 4d). To verify the effect of AC005392.2 on GLUT1, we examined whether AC005392.2 affected the protein stability of GLUT1 in CRC cells using cycloheximide (CHX), an inhibitor of protein translation. Of note, overexpression of AC005392.2 extended the half-life of endogenous GLUT1 protein from 120 min to >480 min in HCT116 cells, while knockdown of AC005392.2 shortened the half-life of GLUT1 protein from >480 min to <120 min in SW620 cells (Figs. 4e and S2c), suggesting that AC005392.2 promotes the stability of GLUT1 protein.

**Table 1:** Mass spectrometry was used to detect the glycolytic molecules pulled down by the AC005392·2 sense and AC005392·2 antisense sequences (45-55 kDa).

Accession	Description		Unique peptides	% Protein coverage
P06733 | ENOA_HUMAN	Alpha-enolase OS = Homo sapiens OX = 9606 GN = ENO1 PE = 1 SV = 2	Sense	20	43
		Anti-sense	25	51
P14618 | KPYM_HUMAN	Pyruvate kinase PKM OS = Homo sapiens OX = 9606 GN = PKM PE = 1 SV = 4	Sense	21	39
		Anti-sense	29	56
P04075 | ALDOA_HUMAN	Fructose-bisphosphate aldolase A OS = Homo sapiens OX = 9606 GN = ALDOA PE = 1 SV = 2	Sense	13	35
		Anti-sense	18	47
P00558 | PGK1_HUMAN	Phosphoglycerate kinase 1 OS = Homo sapiens OX = 9606 GN = PGK1 PE = 1 SV = 3	Sense	20	49
		Anti-sense	26	68
P00338 | LDHA_HUMAN	L-lactate dehydrogenase A chain OS = Homo sapiens OX = 9606 GN = LDHA PE = 1 SV = 2	Sense	None	
		Anti-sense	1	4
P09104 | ENOG_HUMAN	Gamma-enolase OS = Homo sapiens OX = 9606 GN = ENO2 PE = 1 SV = 3	Sense	None	
		Anti-sense	1	8
P11166 | GTR1_HUMAN	Solute carrier family 2 facilitated glucose transporter member 1 OS = Homo sapiens OX = 9606 GN = SLC2A1 PE = 1 SV = 2	Sense	2	4
		Anti-sense	none	

Considering that more than 80% of proteins in human cells are degraded via the ubiquitin-proteasome pathway [21], we analyzed the effect of AC005392.2 on the degradation of GLUT1 using MG132, a proteasome inhibitor. Notably, AC005392.2 overexpression enabled GLUT1 to be maintained at the same level in HCT116 cells following MG132 treatment for the indicated times, while a marked increase in the level of GLUT1 degradation was observed in SW620 cells after depletion of AC005392.2 (Fig. 4f). Consistently, AC005392.2 overexpression decreased the ubiquitination of GLUT1 in HCT116 cells, while AC005392.2 knockdown promoted the ubiquitination and degradation of GLUT1 in SW620 cells (Fig. 4g). These results suggest that AC005392.2 stabilizes GLUT1 by interfering with the ubiquitin-proteasome pathway. A recent study indicated that GLUT1 associated lncRNA (GAL) stabilizes GLUT1 protein by enhancing GLUT1 SUMOylation to inhibit GLUT1 ubiquitination [22]. As expected, we also found that overexpression of AC005392.2 increased GLUT1 SUMOylation, as determined by immunoprecipitation and western blotting, while AC005392.2 knockdown decreased it (Fig. 4h). Furthermore, small ubiquitin-related modifier 1 (SUMO1) expression was reversed in HCT116 and SW620 cells stably transfected with AC005392.2 clone vector or AC005392.2 shRNA (Fig. S2d). The results showed that knockdown of SUMO1 inhibited the AC005392.2-mediated upregulation of GLUT1 levels in HCT116 cells, while overexpression of SUMO1 had little effect on GLUT1 in AC005392.2-knockdown SW620 cells (Fig. 4i), suggesting that SUMO1-induced upregulation of GLUT1 depends on AC005392.2. Altogether, these results suggest that AC005392.2 stabilizes the GLUT1 protein by enhancing GLUT1 SUMOylation, thus inhibiting GLUT1 ubiquitination.

### GLUT1 contributes to SOX2-mediated glycolysis and VM in CRC

**Fig. 5:**
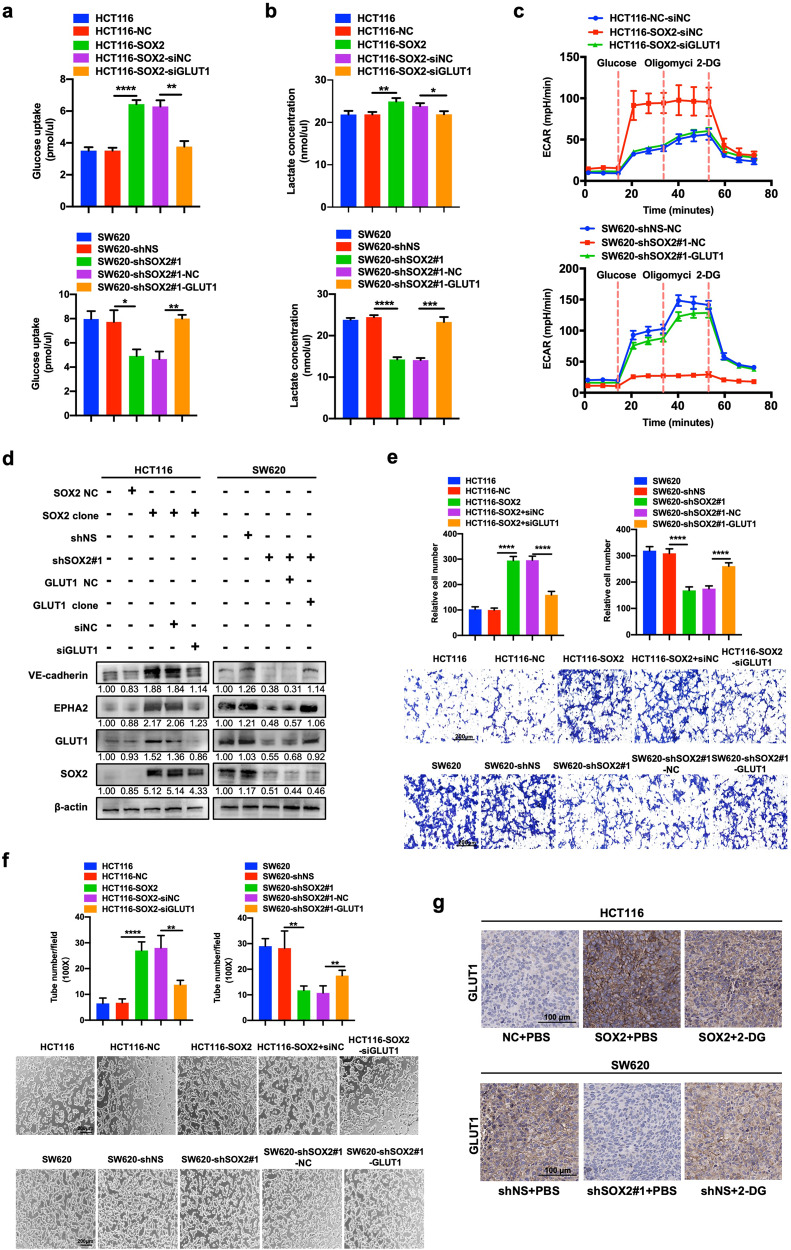
GLUT1 contributes to SOX2-mediated glycolysis and vasculogenic mimicry in CRC. **a**, **b** Glucose consumption (**a**) and lactate production (**b**) were assessed using fluorescence-based kits in HCT116 cells co-transfected with SOX2 clone and GLUT1 siRNA or in SW620 cells co-transfected with SOX2 shRNA and GLUT1 clone for 72 h (mean ± SD; *n* = 3, two-tailed Student’s *t* test). **c** ECAR was examined in transfected HCT116 cells and SW620 cells using a Seahorse XF assay. **d** Western blotting was performed using the indicated antibodies in transfected HCT116 cells and SW620 cells. **e**, **f** Transwell migration assays (**e**) and tube formation assays (**f**) were conducted in transfected HCT116 cells and SW620 cells (Scale, 200 μm; mean ± SD; *n* = 3, two-tailed Student’s *t* test). **g** IHC staining of GLUT1 in SOX2-overexpressing HCT116 and SOX2-knockdown SW620 xenografts. Scale, 100 μm. *p < 0.05, **p < 0.01, ***p < 0.001, and ****p < 0.0001.

GLUT1, a transporter facilitating the uptake of glucose, was reported to regulate VM formation in nasopharyngeal carcinoma [16]. Similarly, we found GLUT1 overexpression dramatically increased glucose consumption, lactate production, and ECAR in HCT116 cells (Figs. S2e and S3a–c), while knockdown of GLUT1 did the opposite in SW620 cells. Moreover, the expression of VM-related molecules and VM formation were also regulated by GLUT1 expression (Fig. S3d–f). These results indicate that GLUT1 can promote glycolysis and VM formation in CRC. To further investigate the involvement of GLUT1 in SOX2-mediated glycolysis and VM formation, GLUT1 expression was reversed in HCT116 and SW620 cells stably transfected with a SOX2 clone vector or SOX2 shRNA (Fig. S2f). The results revealed that reversing GLUT1 expression partly inhibited the promotion of SOX2 on glucose consumption, lactate production, and ECAR (Fig. 5a–c). Most importantly, the expressions of VM-related molecules and VM formation driven by SOX2 were also impaired by GLUT1 dysregulation (Fig. 5d–f). Furthermore, IHC results of xenograft tissues confirmed the effect of SOX2 on GLUT1 expression in vivo (Fig. 5g). Overall, these results demonstrate that GLUT1 is necessary for SOX2-mediated glycolysis and VM in CRC.

### Expression of AC005392.2, GLUT1, and EPHA2 in CRC clinical samples

**Fig. 6:**
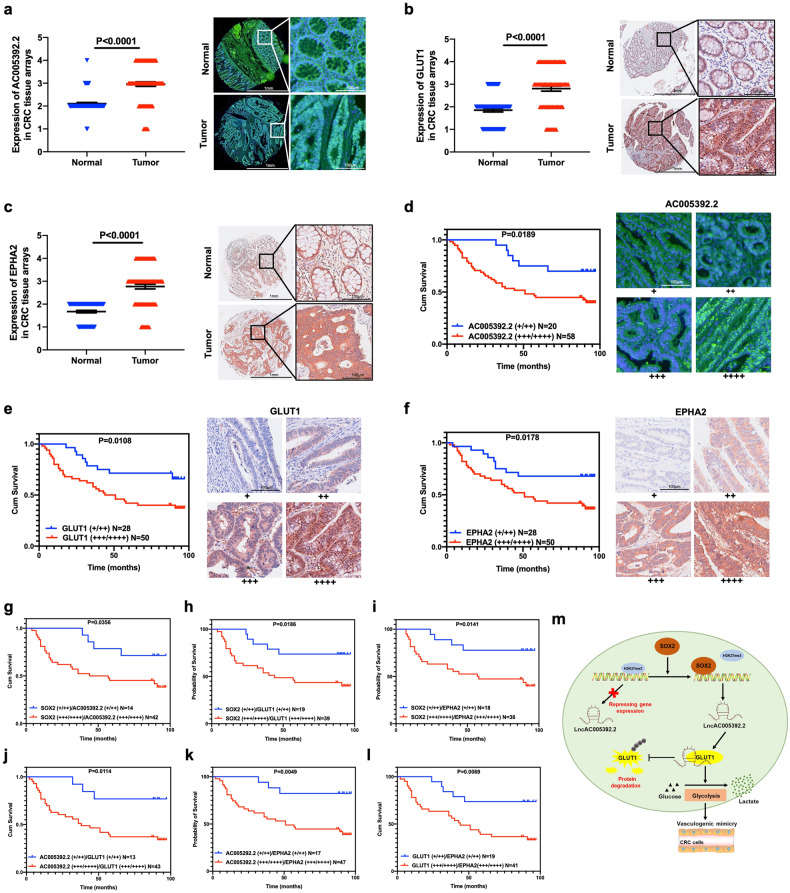
Expression of AC005392.2, GLUT1, and EPHA2 in clinical samples. **a**–**c** The intensity of staining of malignant cells was scored to analyze levels of AC005392.2 (**a**), GLUT1 (**b**), and EPHA2 (**c**) were examined by FISH and IHC in a microarray with 78 pairs of CRC tissues (Scale, 1 mm; inset: scale, 100 μm; mean ± SD; Normal = 74, Tumor = 78, two-tailed Student’s *t* test). Representative sections are shown. **d**–**f** Survival curves were generated using the Kaplan–Meier method (median values as cutoff) according to the expression of AC005392.2 (**d**), GLUT1 (**e**), and EPHA2 (**f**). Representative sections are shown (Scale, 100 μm, *n* = 78, log-rank test). **g**–**i** Survival curves of CRC patients stratified by high or low expression of SOX2 and AC005392.2 (**g**, *n* = 56), GLUT1 (**h**, *n* = 58) or EPHA2 (**i**, *n* = 56) were estimated using the Kaplan–Meier method and compared using the Log-rank test (median values as cutoff, log-rank test). **j**, **k** Survival curves of CRC patients stratified based on high or low expression of AC005392.2 and GLUT1 (**j**, *n* = 56) or EPHA2 (**k**, *n* = 64) were estimated using the Kaplan-Meier method and compared using the Log-rank test (median values as cutoff, log-rank test). **l** Survival curves of CRC patients stratified by high or low expression of GLUT1 and EPHA2 were estimated using the Kaplan–Meier method and compared using the Log-rank test (median values as cutoff, *n* = 60, log-rank test). **m** A schematic model of the molecular mechanism by which SOX2 Promotes VM formation in CRC.

To investigate the role of AC005392.2, GLUT1, and EPHA2 in human CRC progression, a microarray analysis using 78 pairs of CRC tissue was performed. The results showed that higher levels of AC005392.2, GLUT1, and EPHA2 were found in tumor tissues than in surrounding normal tissues (Fig. 6a–c). The Kaplan–Meier survival analysis revealed that higher levels of AC005392.2, GLUT1, or EPHA2 were associated with poor prognosis (Fig. 6d–f). Furthermore, lower levels of both SOX2 and AC005392.2, GLUT1, or EPHA2 resulted in a promising prognosis in patients with CRC (Fig. 6g–i). A similar trend was also observed in patients with lower levels of both AC005392.2 and GLUT1 or EPHA2 (Fig. 6j, k). We consistently found that patients with low levels of both GLUT1 and EPHA2 had a better prognosis (Fig. 6l). Correlation analysis suggested that SOX2 was positively associated with AC005392.2, GLUT1, and EPHA2 in CRC samples (Table 2), while AC005392.2 showed a positive correlation with GLUT1 and EPHA2 (Table 3). Also, GLUT1 and EPHA2 showed a significant positive correlation (Table 4). In summary, these findings imply that the expression of SOX2, AC005392.2, GLUT1, and EPHA2 are noticeably correlated in clinical samples and may predict prognoses in patients with CRC.

**Table 2:** Correlation analysis between SOX2, AC005392·2, GLUT1, and EPHA2.

		SOX2 scoring		Correlation coefficient (*R*)
		Low expression (+/++) (*n* = 30)	High expression (+++/++++) (*n* = 48)	
AC005392·2 Scoring	Low expression (+/++) (*n* = 20)	14	6	*R* = 0.253
	High expression (+++/++++) (*n* = 58)	16	42	*P* = 0.025
GLUT1 Scoring	Low expression (+/++) (*n* = 28)	19	9	*R* = 0.491
	High expression (+++/++++) (*n* = 50)	11	39	*P* = 0.000
EPHA2 Scoring	Low expression (+/++) (*n* = 28)	18	10	*R* = 0.240
	High expression (+++/++++) (*n* = 50)	12	38	*P* = 0.034

**Table 3:** Correlation analysis between AC005392·2, GLUT1, and EPHA2.

		AC005392·2 scoring		Correlation coefficient (*R*)
		Low expression (+/++) (*n* = 20)	High expression (+++/++++) (*n* = 58)	
GLUT1 Scoring	Low expression (+/++) (*n* = 28)	13	15	*R* = 0.376
	High expression (+++/++++) (*n* = 50)	7	43	*P* = 0.001
EPHA2 Scoring	Low expression (+/++) (*n* = 28)	17	11	*R* = 0.283
	High expression (+++/++++) (*n* = 50)	3	47	*P* = 0.012

**Table 4:** Correlation analysis between GLUT1 and EPHA2.

		GLUT1 scoring		Correlation coefficient (*R*)
		Low expression (+/++) (*n* = 28)	High expression (+++/++++) (*n* = 50)	
EPHA2 Scoring	Low expression (+/++) (*n* = 28)	19	9	*R* = 0.437
	High expression (+++/++++) (*n* = 50)	9	41	*P* = 0.000

## Discussion

Vasculogenic mimicry (VM), which was first reported in a highly aggressive uveal melanoma in 1999 [8], is an alternative method of supplying blood, independent of endothelial vessels. This finding provides a new perspective on the blood supply of tumors. To date, VM has been found in a variety of cancer types, including breast cancer [23], ovarian cancer [24], and colorectal cancer [25], and is associated with poor prognoses in patients. It may even explain why anti-VEGF/VEGFR therapy fails, as this treatment would have no effect on VM formation. The development of drugs based on VM theory is predicted to change the field of cancer treatment for patients, however, the mechanism of VM formation remains unclear. Our previous study has shown that SOX2 promotes VM formation in CRC [10]. In this study, we demonstrate that the SOX2-lncRNA AC005392.2-GLUT1 axis is essential for VM formation in CRC. The discovery of this axis may help develop potential biomarkers for diagnosis and therapeutic candidates for treatment.

Understandably, the metabolic switch from mitochondrial respiration to glycolysis is critical for cancer cell growth. High levels of glycolysis result in the accumulation of lactate and succinate, and a reduction in β-hydroxybutyrate, which collectively promote tumor growth and progression [26]. A recent study indicated that glycolysis activation promotes VM formation in nasopharyngeal carcinoma [16]. Consistent with this, we have found that SOX2 promotes VM formation in a glycolysis-dependent manner in CRC. SOX2 increases glucose consumption, lactate production, and the extracellular acidification rate of CRC cells. Blockage of glycolysis using the glycolysis inhibitor 2-DG efficiently attenuates SOX2-induced VM formation in vitro and in vivo. These findings suggest that glycolysis has an essential role in SOX2 promotion of VM in CRC, and targeting glycolysis holds great promise for therapeutic intervention.

A growing number of studies have shown that tumor glycolysis, invasion, and metastasis can be regulated by lncRNAs [14]. Targeting lncRNAs could be a new approach for cancer treatment. In this study, we used a lncRNA microarray to analyze lncRNAs regulated by SOX2, and discovered a new lncRNA, AC005392.2, which is significantly upregulated by SOX2 in CRC. AC005392.2 knockdown markedly restrained SOX2-mediated glycolysis and VM formation, whereas AC005392.2 overexpression rescued the inhibition of glycolysis and VM formation caused by SOX2 depletion, supporting the involvement of AC005392.2 in SOX2-driven malignant CRC phenotypes. We next explored the mechanism by which SOX2 regulates AC005392.2 and found that SOX2 transcriptionally activates AC005392.2 expression via binding with its promoter (−1031 ~ −1041, upstream of TSS). Of particular note, recent studies have shown that aberrant expression of lncRNAs is partially attributed to abnormal epigenetic regulation [18, 19]. We also found that SOX2 reduces H3K27me3 enrichment at the AC005392.2 promoter, which relieves the transcriptional repression of AC005392.2. In addition, we found that AC005392.2 is upregulated in CRC tumor tissues and is predictive of a poor prognosis in patients. AC005392.2 is detected to be positively correlated with SOX2 and VM marker EPHA2 in CRC, and patients with high levels of AC005392.2 and SOX2 or EPHA2 have a worse prognosis than those with low expression. Taken, together, these findings decipher a molecular mechanism of action for AC005392.2 in sustaining the oncogenic functions of SOX2 in CRC.

Screening for lncRNA interacting proteins is crucial to understand the biological functions of lncRNA molecules. Given the striking effect of AC005392.2 in promoting glycolysis, we further investigated the underlying molecular mechanism. RNA pull-down followed by mass spectrometry reveals that AC005392.2 directly binds with GLUT1 protein. Glucose transporters functioning at the first step of glycolysis are responsible for glucose translocation across the cell membrane [27]. Several studies have demonstrated that glucose transporters, such as GLUT1 and GLUT3, exert primary control over glycolytic flux [28]. Consistent with this, we found that overexpression of GLUT1 enhances glycolysis in CRC cells, while depletion of GLUT1 impairs SOX2-driven glycolysis and VM formation. This supports the idea that the SOX2-lncRNA AC005392.2-GLUT1 signaling axis is important in malignant CRC phenotypes. LncRNAs are reported to participate in various physical and pathological processes by acting as scaffolds, guides, decoys or repressors, activators, and sponges at the transcriptional, post-transcriptional or epigenetic levels [29]. In this study, we validated that AC005392.2 is localized in the cytoplasm of CRC cells and exerts post-transcriptional regulation. AC005392.2 binds with GLUT1 and contributes to GLUT1 protein stability by interfering with the ubiquitin-proteasome system. Furthermore, a recent study reported that SUMOylation enables stabilization of GLUT1 protein by inhibiting the ubiquitin-proteasome system [22]. Consistent with this report, our findings reveal that AC005392.2 stabilizes GLUT1 protein by enhancing GLUT1 SUMOylation to inhibit GLUT1 ubiquitination and degradation in CRC. Additionally, elevated GLUT1 is observed in CRC tumor tissues and associated with shorter survival in CRC patients. GLUT1 is positively associated with SOX2, AC005392.2, and EPHA2, and their upregulation leads to a poor prognosis in CRC patients.

In summary, because glycolysis and VM formation are prevalently dysregulated in cancers, therapeutic interventions based on the SOX2-lncRNA AC005392.2-GLUT1 axis may be promising in the treatment of CRC patients. Our data suggest that SOX2 promotes VM formation in a glycolysis-dependent manner. SOX2 effectively enhances glycolysis by transcriptionally activating AC005392.2 expression, which stabilizes GLUT1 protein by increasing SUMOylation to inhibit ubiquitination and degradation of GLUT1 (Fig. 6m). Thus, our study uncovers a new mechanism of VM formation in CRC, offering novel therapeutic clues for the development of new antiangiogenic drugs or new combination regimens.

### Supplementary information

Reproducibility Checklist

Supplementary figure and table legends

Supplementary Figure S1

Supplementary Figure S2

Supplementary Figure S3

Supplementary Table S1

Supplementary Table S2

Supplementary Table S3

Supplementary Table S4

Detailed methods

Original western blots

## Data Availability

The accession number for the Gene set enrichment analysis (GSEA) data reported in this paper can be found under GEO: GSE17538. Source data for Gene Ontology (GO) analysis are provided in Table S3. Source data for lncRNA microarray analysis are provided in Table S4. Original western blots are provided in Supplementary Material. Further information and requests for resources and reagents should be directed to and will be fulfilled by the lead contact, HZ (haoxuan.zheng@qq.com).
