# Supplementary material for: SOX2 promotes vasculogenic mimicry by accelerating glycolysis via the lncRNA AC005392.2-GLUT1 axis in colorectal cancer
Source: Cell Death Dis. 2023 Dec 4;14(12):791. doi: 10.1038/s41419-023-06274-1 (PMC10694132; doi:10.1038/s41419-023-06274-1)
Supplement: Supplementary file 2 — Supplementary figure and table legends [file 41419_2023_6274_MOESM2_ESM.docx]

**Supplementary figure legends**

**Fig. S1. Glycolysis is involved in SOX2-induced vasculogenic mimicry in CRC cells.**

**a,** Scattergram of GO enrichment analysis for differentially expressed genes (DEGs, FDR < 0.05) in HCT116 cells after transfection with SOX2 clones. **b,** mRNA and protein level of SOX2 were examined in the indicated CRC cells. **c,** HCT116 and SW620 cells were transfected with a SOX2 clone or SOX2 shRNA for 48 h. qRT-PCR was performed to analyze the expression of the indicated molecules (mean ± SD; n=3, two-tailed Student’s t-test). **d,** HCT116 and SW620 cells were transfected with SOX2 clone or SOX2 shRNA for 72 h, then treated with 2-DG (3 mM) for 24 h. Transwell migration assays were performed to assess the migratory ability of the indicated cells (Scale, 200μm; mean ± SD; n=3, two-tailed Student’s t-test). **e,** Western blotting was performed to analyze the indicated proteins in SOX2-overexpressing HCT116 and SOX2-knockdown SW620 xenografts. **p* < 0.05, ***p* < 0.01, ****p* < 0.001 and *****p* < 0.0001.

**Fig. S2. The efficacy of genes transfection.**

**a** and **b,** Overexpression or knockdown of AC005392.2 was performed in HCT116 (a) or SW620 (b) cells transfected with SOX2 clone or SOX2 shRNA. Gene transfection efficacy was evaluated using qRT-PCR (mean ± SD; n=3, two-tailed Student’s t-test) or western blotting. **c,** HCT116 and SW620 cells were transfected with AC005392.2 clone or AC005392.2 shRNA for 48 h. The expression of AC005392.2 was assessed by qRT-PCR (mean ± SD; n=3, two-tailed Student’s t-test). **d,** Overexpression or knockdown of SUMO1 was conducted in HCT116 or SW620 cells transfected with AC005392.2 clone or AC005392.2 shRNA. Gene transfection efficacy was evaluated by qRT-PCR (mean ± SD; n=3, two-tailed Student’s t-test) or western blotting at 48 h and 72 h after transfection, respectively. **e,** HCT116 and SW620 cells were transfected with GLUT1 clone or GLUT1 siRNA. The expression of GLUT1 was assessed at 48 h and 72 h by qRT-PCR (mean ± SD; n=3, two-tailed Student’s t-test) or western blotting, respectively. **f,** Overexpression or knockdown of GLUT1 was conducted in HCT116 or SW620 cells transfected with SOX2 clone or SOX2 shRNA. Gene transfection efficacy was evaluated by qRT-PCR (mean ± SD; n=3, two-tailed Student’s t-test) or western blotting. **p* < 0.05, ***p* < 0.01, ****p* < 0.001 and *****p* < 0.0001.

**Fig. S3. GLUT1 is involved in CRC glycolysis and vasculogenic mimicry.**

**a** and **b,** HCT116 and SW620 cells were transfected with a GLUT1 clone or GLUT1 siRNA for 72 h. The effect of GLUT1 on glucose consumption (a) and lactate production (b) was assessed using fluorescence-based kits (mean ± SD; n=3, two-tailed Student’s t-test). **c,** The effect of GLUT1 on the extracellular acidification rate (ECAR) was measured using a Seahorse XF assay in HCT116 and SW620 cells. **d,** HCT116 and SW620 cells were transfected with a GLUT1 clone or GLUT1 siRNA for 72 h. Western blotting was performed using the indicated antibodies. **e** and **f,** HCT116 and SW620 cells were transfected with a GLUT1 clone or GLUT1 siRNA for 72 h. Transwell migration assays (e) and tube formation assays (f) were conducted (Scale, 200μm; mean ± SD; n=3, two-tailed Student’s t-test). **p* < 0.05, ***p* < 0.01, ****p* < 0.001 and *****p* < 0.0001.

**Supplementary table legends**

**Table S1.** Key resources table.

**Table S2.** List of proteins pulled down by AC005392.2 sense and antisense probe at the size of 45-55kDa.

**Table S3.** Gene Ontology (GO) analysis of SOX2-overexpressing HCT116 cells and its negative control.

**Table S4.** List of differentially expressed lncRNAs in SOX2-overexpressing HCT116 cells and its negative control.
