# Supplementary figures and images for: SOX2 promotes vasculogenic mimicry by accelerating glycolysis via the lncRNA AC005392.2-GLUT1 axis in colorectal cancer
Source: Cell Death Dis. 2023 Dec 4;14(12):791. doi: 10.1038/s41419-023-06274-1 (PMC10694132; doi:10.1038/s41419-023-06274-1)

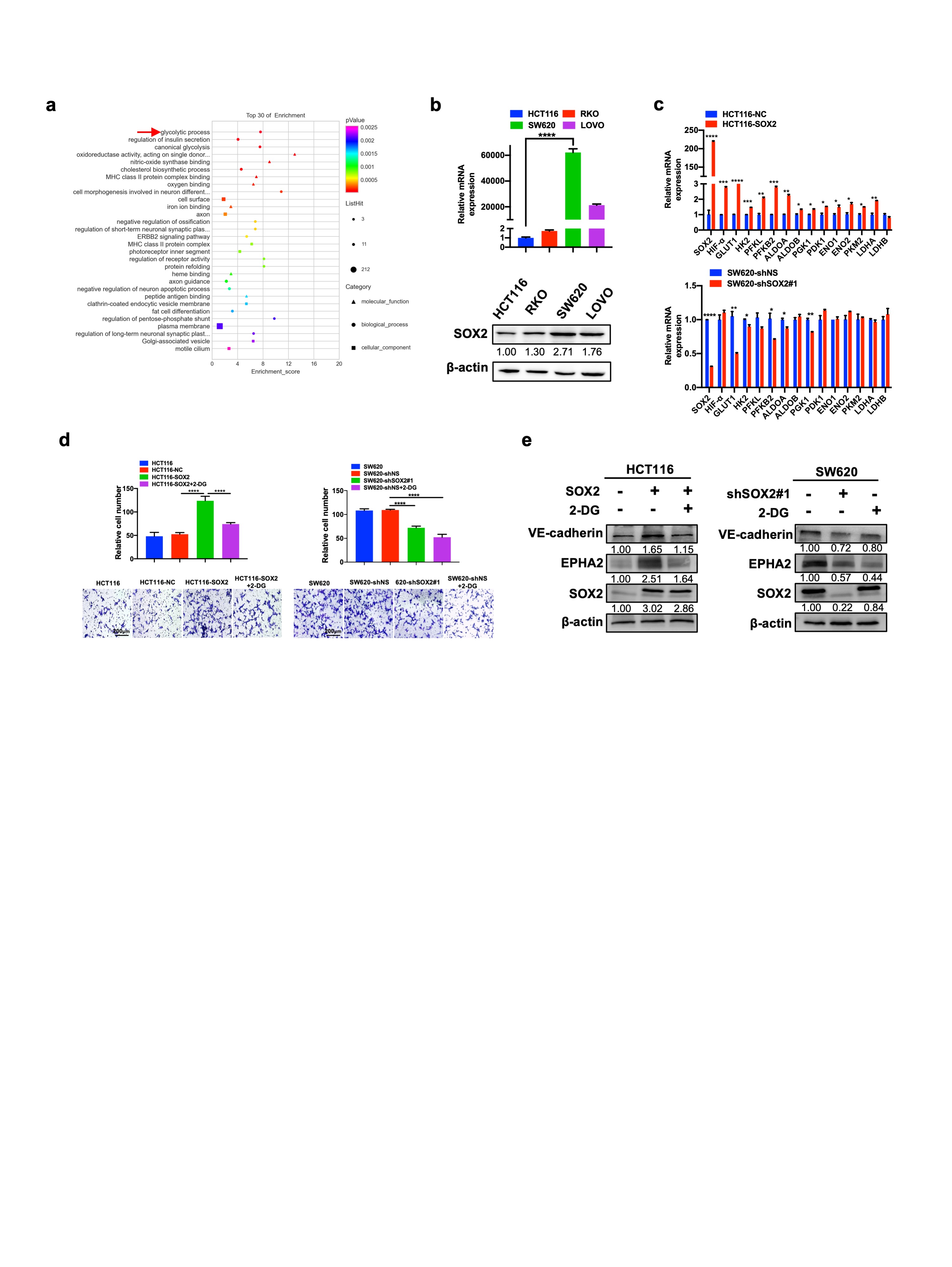

Supplement: Supplementary file 3 — Supplementary Figure S1 [file 41419_2023_6274_MOESM3_ESM.jpg]

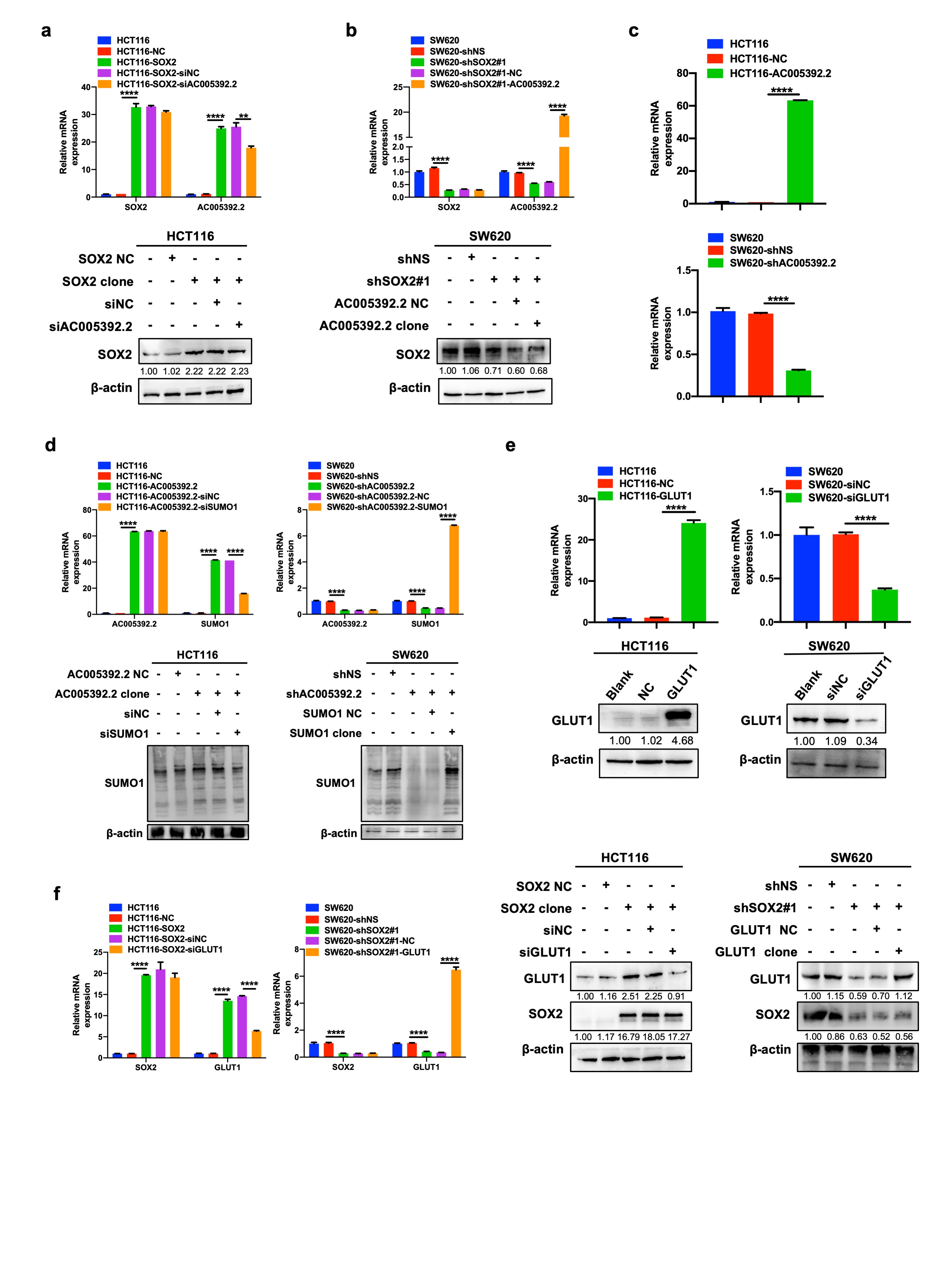

Supplement: Supplementary file 4 — Supplementary Figure S2 [file 41419_2023_6274_MOESM4_ESM.jpg]

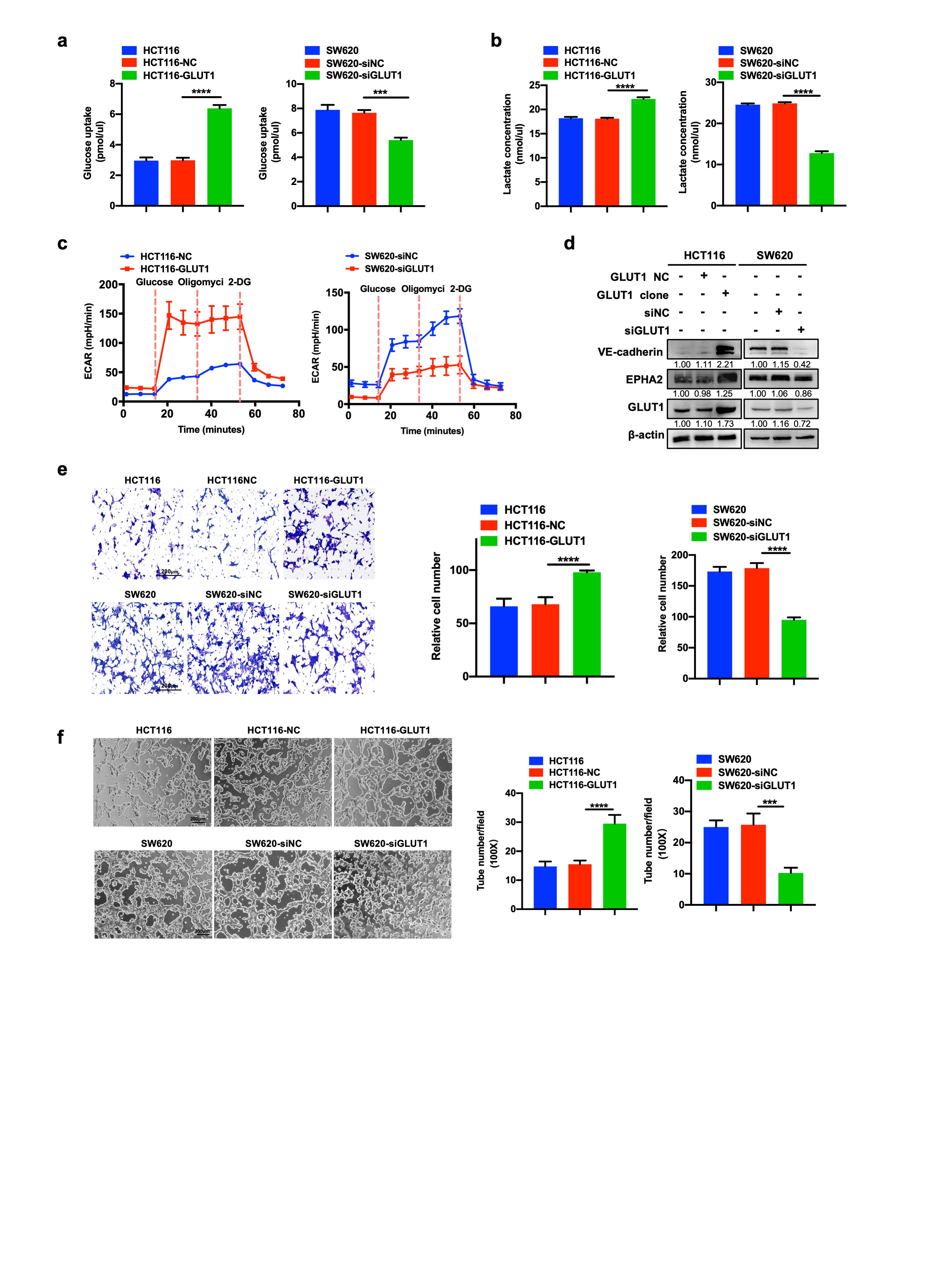

Supplement: Supplementary file 5 — Supplementary Figure S3 [file 41419_2023_6274_MOESM5_ESM.jpg]
