# Supplementary material for: SOX2 promotes vasculogenic mimicry by accelerating glycolysis via the lncRNA AC005392.2-GLUT1 axis in colorectal cancer
Source: Cell Death Dis. 2023 Dec 4;14(12):791. doi: 10.1038/s41419-023-06274-1 (PMC10694132; doi:10.1038/s41419-023-06274-1)
Supplement: Supplementary file 10 — Detailed methods [file 41419_2023_6274_MOESM10_ESM.docx]

**Materials and Methods**

**Cell Lines**

Human CRC cell lines, HCT116 and SW620 were purchased from the American Type Culture Collection (ATCC) and authenticated according to the recommendations of ATCC. Cells were grown in RPMI 1640 (Gibco, C11875500BT) for HCT116 cells or DMEM (Gibco, 11995040) for SW620 cells. Media was supplemented with 10% fetal bovine serum (Gibco, 10099-141) in an incubator with 5% CO_2_ at 37°C.

**Cell Transfection, Quantitative real-time polymerase chain reaction (qRT-PCR) and Western Blotting**

SW620 and HCT116 cells (1×10^5^) were seeded into 6-well plates and grown to ~70% confluence. Vectors were transfected into cells using a Lipofectamine 3000 reagent (Invitrogen, L3000015) according to the manufacturer’ protocols. Lentivirus was purchased from Shanghai Genechem Co. Ltd. Overexpression plasmid and siRNA were purchased from Guangzhou Youming Biotechnology or Guangzhou Kidan Biotechnology. All sequences are listed in Table S1. qRT-PCR and western blotting were used to detect the RNA and protein expression of each indicator in this study. Assays were performed as previously described(1). The primers for qRT-PCR and the antibodies for western blotting are listed in Table S1.

**Three-dimensional (3D) Tube Formation and Transwell Migration assays**

For 3D tube formation assay, VM formation was tested using a 3D culture containing Matrigel (BD, USA) in vitro. Culture plates with 24 wells were coated with Matrigel (100 μl/well). The CRC cells were trypsinized and suspended in the complete medium at 3 × 10^5^ cells/ml, transferred onto the surface of Matrigel at 1 ml/well, and incubated at 37°C for 48 h. The number of tube-like structures was calculated under the light microscope. Transwell migration assay was performed, as previously described(1).

**Animal tumor model and in vivo imaging**

HCT116 and SW620 cells were stably transfected with a SOX2 clone vector or SOX2 shRNA (4×10^6^ cells in 100 µl PBS). Transfected cells were subcutaneously injected into 5-week-old female BALB/c nude mice. Seven days later, mice bearing tumors were administered intraperitoneal injections of PBS or the glycolysis inhibitor, 2-Deoxy-D-glucose (2-DG) (0.5g/kg), twice a week. Tumor volumes (mm^3^) were assessed using calipers and calculated every three days throughout the experiment according to the formula (length × width^2^)/2. At the experimental endpoint, mice were used for ^18^F-Fluorodeoxyglucose (^18^F-FDG) Positron emission tomography (PET) imaging assay, and tumors were harvested and weighed. Excised tissues were fixed in 10% neutral-buffered formalin and used for histologic examination. For ^18^F-FDG PET imaging assay, mice were fasted for 8 h and injected with approximately 250 µCi of ^18^F-FDG via lateral tail vein. 50 min later, mice were anesthetized using isoflurane and assessed using micro-PET and micro-CT imaging. ^18^F-FDG uptake was quantified by drawing region of interest (ROI) and plotting maximum standard uptake value (SUVmax). IHC staining and CD31/PAS double-staining assays were performed as previously described(2). For the FISH assay, the specific AC005392.2 probes labeled with green fluorescence were provided by Tsingke Biotechnology Co., Ltd. and detected by FISH Tag™ RNA Multicolor Kit (Thermo Fisher Scientific, F32956). All experimental procedures using animals were conducted as per the animal protocol approved by the Animal Care and Use Committee of Southern Medical University.

**Chromatin Immunoprecipitation (ChIP), Coimmunoprecipitation (co-IP) and** **Luciferase Reporter assays**

ChIP and co-IP assays were performed as previously described(3). For luciferase reporter assays, the AC005392.2 promoter reporter (Guangzhou Youming BioTechnology Co. Ltd.) was introduced into cultured cells using Lipofectamine 3000 (Invitrogen, L3000015), according to the manufacturer’s instructions. After 48 h, cells were lysed, and luciferase activity was assessed with Duo-Lite Luciferase Assay System (Vazyme, DD1205-01), as per the manufacturer’s instructions.

**Biochemical assays**

HCT116 and SW620 cells were plated in 6-well plates (2×10^5^ cells/well) and cultured in RPMI 1640 or DMEM for 24 h. The levels of lactate in the media were quantitated using a fluorescence-based lactate assay kit (BioVision, K607-100). For the glucose uptake assay, cells were seeded into a 96-well plates (1500 cells/well) and cultured for 4 days, before starvation in 100 µl of serum-free RPMI 1640 or DMEM overnight. The level of intracellular glucose was assessed using a fluorescence-based glucose assay kit (BioVision, K676-100). The extracellular acidification rate (ECAR) was measured using the Seahorse XF Glycolysis Stress Test Kit (Agilent Technologies, 103020-100). Briefly, cells were seeded into XF96-well culture plates (10^4^ cells/well) and incubated overnight. Measurements were conducted according to the manufacturer’s instructions. Seahorse Wave software was used to analyze the data.

**RNA Pulldown, Silver-staining and** **Mass Spectrometry (MS)**

RNA pulldown was performed using the PierceTM Magnetic RNA-Protein Pull-Down Kit (Thermo Fisher Scientific, 20164), according to the manufacturer's instructions. The products of RNA pulldown underwent electrophoresis by 10% SDS-PAGE gels, which were then stained using silver (Beyotime, P0017S). The specific stripes in the AC005392.2 sense group and the AC005392.2 antisense group were cut and analyzed by mass spectrometer, provided by Wininnovate Biotech Technology Company. MS based on Silver-stained gel was performed (Wininnovate, Shenzhen, China). IDA (information-dependent acquisition) mass spectrometry techniques were used to acquire tandem MS data on a ThermoFisher Q Exactive mass spectrometer (ThermoFisher, USA) fitted with a Nano Flex ion source. Data were acquired using an ion spray voltage of 1.9 kV, and an interface heater temperature of 275℃. The MS was operated with FULL-MS scans. For IDA, survey scans were acquired in 250 ms and up to 20 product ion scans (50 ms) were collected. Only spectra with a charge state of 2–4 were selected for fragmentation by higher-energy collision energy. The dynamic exclusion rate was set to 25 s. The MS/MS data were analyzed for protein identiﬁcation and quantiﬁcation using PEAKS Studio 8.5. The local false discovery rate at PSM was 1.0% after searching against human database with a maximum of two missed cleavages. The following settings were selected: Oxidation (M), Acetylation (Protein N-term), Deamidation (NQ), Pyro-glu from E, Pyro-glu from Q for variable modifications as well as fixed Carbamidomethylation of cysteine. Precursor and fragment mass tolerance were set to 10 ppm and 0.05 Da.

**RNA Immunoprecipitation (RIP) assay**

The RIP assay was performed using a Magna RIP™ RNA-Binding Protein Immunoprecipitation Kit (Millipore, 17-700). Cells were lysed in lysis buffer containing protease and ribonuclease inhibitors for 30 min on ice and then centrifuged. IgG and anti-GLUT1 antibodies and magnetic beads were added to the remaining supernatant. After incubation overnight, magnetic beads were washed with RIP Wash Buffer and aggregated using a magnetic separator. The supernatant acted as the input positive control. RNA was isolated from the precipitate and detected by qRT-PCR.

**RNA Stability and Protein Stability assays**

Cells were treated with the inhibitor of gene transcription, Act-D (5 μg/ml), or the inhibitor of protein synthesis, CHX (20 μg/ml) for the indicated times. The stability of the isolated RNA or protein was analyzed using qRT-PCR and western blotting, respectively.

**Nuclear Cytoplasmic Fractionation**

Cells were lysed on ice for 10 min using a NE-PER Nuclear and Cytoplasmic Extraction Reagent kit (ThermoFisher Scientific, 78833). The cytoplasmic and nuclear fractions were separated into supernatant and precipitate components, respectively, with a 5-min centrifugation at 500 g. The above-obtained RNA was extracted and subsequently analyzed using qRT-PCR. β-actin and U6 were adopted as cytoplasmic and nuclear markers, respectively.

**Ubiquitination assay**

Cells were treated with 20 μM MG132 for 8 h and lysed in IP lysis/wash buffer. The immune complex was prepared using anti-GLUT1 or control IgG antibodies and was subsequently captured using the Pierce Classic IP Kit (Thermo Fisher, 88804). Subsequently, the isolated samples were analyzed by western blotting with an anti-ubiquitin antibody.

**Tissue Microarray**

Glass-slide tissue arrays including 78 pairs of CRC tissues were purchased from the Shanghai Outdo Biotech (Shanghai, China), and either IHC or FISH assays were performed using the tissue microarray slides. The intensity of staining of malignant cells was scored as follows to analyze the level of protein expression: + (no staining), ++ (weak staining), +++ (moderate staining), and ++++ (strong staining). A score of > ++ was classified as high expression, whereas ≤ ++ was considered as low expression.

**Statistics and Reproducibility**

Statistical analyses were performed using GraphPad Prism 8.0 software and IBM SPSS Statistics software. An unpaired two-tailed Student’s t-test was used to compare two groups. Statistical significance between three or more groups was calculated using a two-way ANOVA. The Wilcoxon matched-pairs signed rank test was used to calculate the difference in the expression of each molecule for ranked datasets. Survival data were plotted as Kaplan–Meier curves and the significance was estimated by Log-rank test. The correlation analysis was assessed using the Pearson method. All data are presented as mean ± SD. A p-value (two-sided)<0.05 was considered statistically significant. **p*<0.05, ***p*<0.01, ****p*<0.001, and *****p*<0.0001.

**Reference**

1. Zheng HX, Cai YD, Wang YD, Cui XB, Xie TT, Li WJ, et al. Fas signaling promotes motility and metastasis through epithelial-mesenchymal transition in gastrointestinal cancer. Oncogene. 2013;32(9):1183-92.

2. Chen J, Chen S, Zhuo L, Zhu Y, Zheng H. Regulation of cancer stem cell properties, angiogenesis, and vasculogenic mimicry by miR-450a-5p/SOX2 axis in colorectal cancer. Cell Death Dis. 2020;11(3):173.

3. Zhu Y, Huang S, Chen S, Chen J, Wang Z, Wang Y, et al. SOX2 promotes chemoresistance, cancer stem cells properties, and epithelial-mesenchymal transition by β-catenin and Beclin1/autophagy signaling in colorectal cancer. Cell Death Dis. 2021;12(5):449.
